# A novel temporary immersion bioreactor system for large scale multiplication of banana (Rasthali AAB—Silk)

**DOI:** 10.1038/s41598-021-99923-4

**Published:** 2021-10-13

**Authors:** Subbaraya Uma, Raju Karthic, Sathiamoorthy Kalpana, Suthanthiram Backiyarani, Marimuthu Somasundaram Saraswathi

**Affiliations:** 1 ICAR-National Research Centre for Banana, Thogamalai Main Road, Thayanur Post, Tiruchirappalli, Tamil Nadu 620 102 India; 2 Bioreactor Facility, Crop Improvement Division, ICAR-National Research Centre for Banana, Thayanur, Tiruchirappalli, Tamil Nadu 620 102 India

**Keywords:** Biological techniques, Biotechnology, Plant sciences

## Abstract

*Musa* sp. cultivar Rasthali (Silk AAB) is a choice variety of the Asian sub-continent. Its production and sustenance are threatened by Fusarium wilt, which affects the livelihoods of small and marginal farmers. The use of quality planting material is one of the strategies to manage the disease. Availability of quality planting material for varieties other than Grand Naine is limited. Large-scale micropropagation using existing technologies is laborious and expensive. Temporary immersion bioreactor system is emerging as a potential advancement in the micropropagation industry. In this study, a cost-effective temporary immersion bioreactor (TIB) system has been developed and an efficient micropropagation method has been standardized. Explants cultured in TIB with 250 ml of culture medium in a 2-min immersion frequency of 6 h were found to be efficient for shoot proliferation and rooting. Its efficacy has been compared with the semisolid culture method. At the end of the 6th subculture, 1496 ± 110 shoots per explant were obtained in TIB. Chlorophyll, carotenoid, stomatal index, and the number of closed stomata were examined to determine the physiological functions of the plants grown in TIB and compared with semisolid grown plantlets. Plantlets grown in TIB were genetically stable and were confirmed using inter-simple sequence repeat (ISSR) markers. The multiplication of shoots in TIB was 2.7-fold higher than the semisolid culture method, which is suitable for large-scale production of planting material for commercial applications.

## Introduction

The Silk subgroup bananas produce unique mealy textured sweet fruits, considered as a low-cost staple food crop cultivated by large proportion of small land holding farmers in India. They are referred by different names across the globe, in India (Rasthali, NanjangudRasabale, Sabri), Bangladesh (Malbhog), Myanmar (Hta-bat), Sri Lanka (Kolikutt),Malaysia (PisangRastali), Philippines (Latundan), Vietnam (ChuoiGoong), Thailand (KluaiNam), Papua New Guinea (Worodong), Hawai ‘i (Manzano, Amorosa, Lady Finger), Australia (Sugar, Lady Finger), Florida (Apple), West Indies (Silk Fig), Tanzania (Pukusa), East Africa (Kipukusu), Latin America (Manzano), Brazil (Maça), etc^1–3^. These Silk bananas are susceptible to *Fusarium* wilt both race 1 and TR4, which affects the production, productivity and livelihood of farming community constantly^4–7^. *Fusarium* wilt, most destructive diseases of banana worldwide, caused by soil-borne fungus *Fusarium oxysporum*f. sp. *cubense* (*Foc*). It is spread through infected plant material and contaminated soil, which wiped out the Gros Michel banana industry in Central America during1950s. Presently, Cavendish cultivars are largely affected by a new strain of *Foc*- tropical race 4 which has spread from Southeast Asia to South Asia, Middle East, and African and Latin American countries, due to lack of effective management strategies^5,6^. Once a field infected by *Foc*, the spores survive in the soil for more than 20 years^5^. Thus, more emphasis should be given to ensure a *Foc* free banana cultivation. Banana being a vegetatively propagated crop, farmers depend on the natural regeneration of side suckers for the supply of planting materials. Infected planting material start showing symptoms from fourth month onwards and eventual crop loss^8,9^. The production of disease-free quality planting material is one of the main strategies to manage the spread.

Micropropagation was the first biotechnological method exploited commercially for the production of disease free, genetically uniform planting material in banana^10^. This technique was standardized for a wide range of *Musa* cultivars belonging to various ploidy and genomes^11^. The success of this technique depends on various factors including explant selection, culture medium and culture conditions^12–14^. The high production cost prevents small- or medium-scale laboratories with limited resources from accessing the potential benefits of plant tissue culture technology^15^. Recent studies have been proposed to utilize automated temporary immersion systems (TIS), which is less expensive and provides an optimal environment for in vitro plant cell, tissue and organ cultures with good productivity^16–18^. Over the years, several temporary immersion systems have been developed and successfully applied for in vitro propagation of banana^19–25^. Till date, reports on Silk bananas are meagre which have high commercial value in developing countries and significant contribution in local economy. This team has developed a temporary immersion bioreactor (TIB)made up of borosilicate glass which can be customized and fabricated as per the culture requirements. This experiment is to study the efficiency of new bioreactor for large-scale propagation of cultivar Rasthali (Silk AAB).

## Results

### Optimization of culture parameters for shoot multiplication in TIB system

The 3rd sub-cultured shoot tip propagules from the semi-solid (SS) medium were used as the initial inoculum for the TIB system to optimize the frequency of immersion, the volume of the culture medium, and inoculum density. Experiments were conducted independently with six replications to standardize each parameter and observations were taken 21 days after the initiation of inoculum (Table 1). To standardize the frequency of immersion, the explants were exposed to liquid media for 2 min for every 4 h, 6 h and 12 h interval. A significant number of shoots per explant was obtained in the immersion frequency of once in 6 h (24.2 shoots/explant), followed by 12 h intervals (18.6 shoots/explant). Similarly, to optimize the volume of media required for the TIB culture system, 100 ml, 250 ml and 500 ml of the liquid medium was used and the maximum number of shoots per explant was observed in 250 ml medium (24 shoots) followed by 500 ml and 100 ml with no significant differences between them. The number of explants required to produce maximum number of shoots have been standardized using various inoculum densities (3, 6 and 12) per system. An average of 24 shoots/explant was obtained from the system with the explant density of 3 and 6 followed by 12. The efficiency of shoot formation in TIB system was enhanced in the range of 1.8 to 2.7 times (16.6 to 24.4 shoots per explant) than the existing shoot tip culture in SS medium (9 shoots per explants). This result revealed that the optimum culture condition for the TIB system for shoot multiplication is: 250 ml of medium, 6 h immersion frequency with 6 explants/bioreactor.

**Table 1:** Effect of the culture systems on immersion frequency, volume of culture medium and inoculum density on multiplication shoots per explant.

Culture system	Mean number of shoots per explant ± SD
Semi solid culture	9.0 ± 1.3^e^
**TIB- Immersion frequencies (2-min immersion)**^**1**^	
4 h	16.6 ± 1.0^d^
6 h	24.2 ± 1.0^a^
12 h	18.6 ± 0.6^c^
**Volume of culture medium (ml)**^**2**^	
100	22.3 ± 0.5^b^
250	24.0 ± 0.6^a^
500	22.6 ± 0.5^b^
**Inoculum density (number of explants cultured/bioreactor)**^**3**^	
3	24.0 ± 0.4^a^
6	24.4 ± 0.5^a^
12	22.6 ± 1.0^b^

Results were recorded after 21 days of culture duration. Numbers represent the mean ± SD (standard deviation). Means with different letter are significantly different (DMRT, p ≤ 0.05).

^1^Experiment carried out with 250 ml of culture medium and 6 numbers of inoculum used.

^2^Experiment carried out with 2-min immersion for every 6 h and 6 numbers of inoculum used.

^3^Experiment carried out with 250 ml of culture medium and 2-min immersion for every 6 h. Data shown in the table are recorded during the 3rd subculture of the study.

### Efficiency of TIB system on shoot multiplication

**Figure 1:**
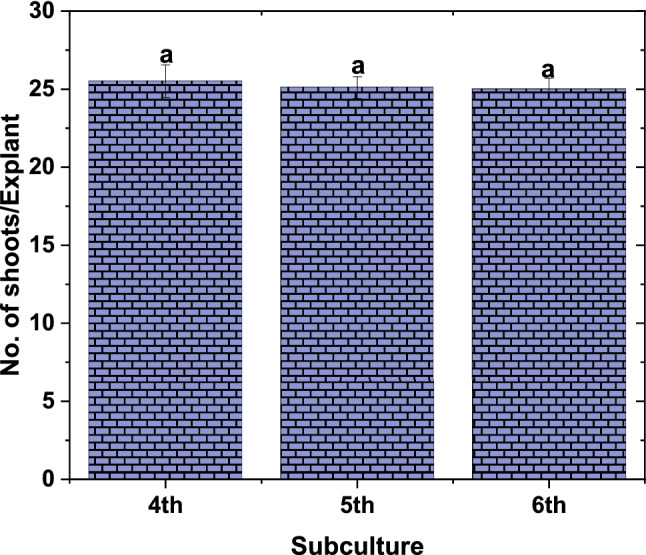
Effect of subcultures on production of multiple shoots in TIB. Experiment carried out with 250 ml of culture medium and 6 numbers of inoculum used and 2-min immersion for every 6 h. results were recorded after 21 days of culture period. (Graph was generated using OriginLab Professional Version 2021b software; URL link: www.OriginLab.com/2021b).

For large scale multiplication of shoots, three more subcultures were carried out in the TIB system with the optimized protocol by splitting shoot clusters up to 2 to 4 portions (2 cm^3^ used as explant)**.**Number of shoots produced per explant was recorded at the time of each subculture and found that there is no significant difference between the sub cultures. An average of 24 and 9 shoots were produced in each explant per subculture in TIB and SS system respectively (Fig. 1). At the end of 6th subculture, 1496 ± 110 shoots per explant were obtained in TIB.

### Optimization of culture parameters for rootingin TIB system

To optimize the immersion frequency and volume of the rooting medium, the proliferated shoot clusters in TIB were replaced with the rooting medium. TIB system performed well for shoot elongation, rooting percentage, and root length than the cultures grown in SS medium. It was observed that shoot height was significantly higher in the TIB system in all the parameters tested than SS system. The root length was significantly high in immersion frequency of 12 h followed by 6 h, then semi-solid grown plants. However, the root length was not affected by the volume of the medium used. The rooting percentage and the number of roots per shoot were not influenced by the immersion frequency and volume of the culture medium, but it is significantly higher in TIB than SS system. These results revealed that 250 ml of rooting medium with the immersion frequency at 6hinterval is optimum for rooting in TIB (Table 2).

**Table 2:** Effect of the culture system, immersion frequency, and volume of culture medium on rooting, and shoot elongation.

Culture system	Rooting (%)	No. of roots/shoot	Root length (cm)	Shoot height (cm)
Semi solid culture	94.3 ± 1.5^c^	5.65 ± 0.7^a^	9.65 ± 1.0^d^	09.25 ± 0.2^d^
**TIB-Immersion frequencies (2-min immersion)**^**1**^				
4 h	97.6 ± 1.7^ab^	6.0 ± 1.0^a^	10.3 ± 1.0^cd^	11.1 ± 0.6^c^
6 h	98.6 ± 1.0^a^	6.5 ± 0.7^a^	12.0 ± 1.1^b^	16.4 ± 0.9^a^
12 h	96.1 ± 2.0^abc^	6.3 ± 0.8^a^	14.4 ± 0.8^a^	17.8 ± 1.9^a^
**Volume of culture medium (ml)**^**2**^				
100	95.3 ± 2.7^bc^	5.5 ± 0.9^a^	12.7 ± 1.5^b^	14.5 ± 1.8^b^
250	98.6 ± 1.1^a^	6.3 ± 0.4^a^	11.5 ± 1.2^bc^	17.5 ± 1.4^a^
500	96.4 ± 2.2^abc^	6.0 ± 1.1^a^	12.8 ± 1.4^b^	17.7 ± 1.7^a^

Results were recorded after 84 days of culture duration. Numbers represent the mean ± SD. Means with different letter are significantly different (DMRT, p ≤ 0.05).

^1^Experiment carried out with 250 ml of culture medium and 150 numbers of inoculum used.

^2^Experiment carried out with 2-min immersion for every 6 h and 150 numbers of inoculum used.

### Physio-chemical changes of culture medium

**Figure 2:**
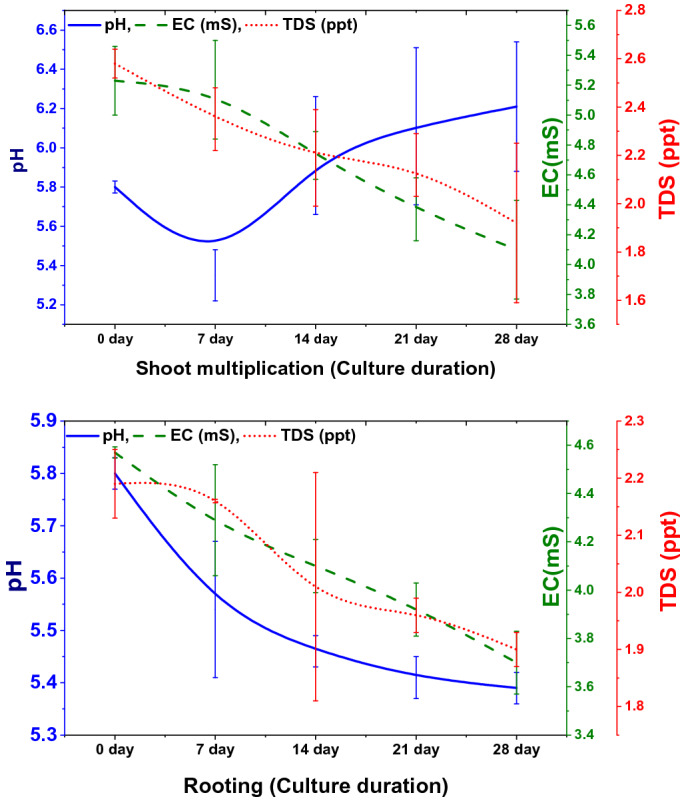
Physio-chemical changes in the media during the culture period in TIB (Graphs were generated using OriginLab Professional Version 2021b software; URL link: www.OriginLab.com/2021b).

The change in the physicochemical parameters (pH, EC and TDS) were recorded during the culture period in the shooting and rooting medium of the TIB system. A sudden decrease in pH (from 5.8 to 5.4) was recorded during 0–7 days of initiation, which was gradually increased thereafter and reached 6.2 on 28th day of inoculation. However, a gradual decrease in EC and TDS was recorded upon prolonged culture duration. Unlike shooting medium, the pH, EC and TDS were gradually decreased in the rooting medium (Fig. 2).

### Effect of culture systems on acclimatization of plantlets in hardening

**Figure 3:**
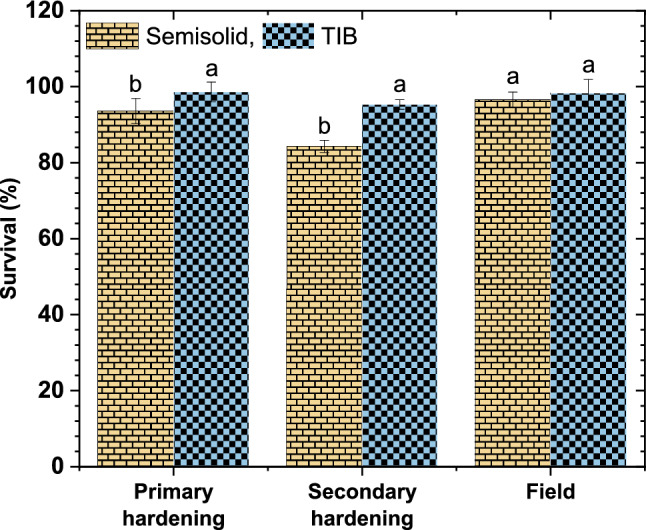
Effect of culture system on survival of plantlets in acclimatization. (Graph was generated using OriginLab Professional Version 2021b software; URL link: www.OriginLab.com/2021b).

**Figure 4:**
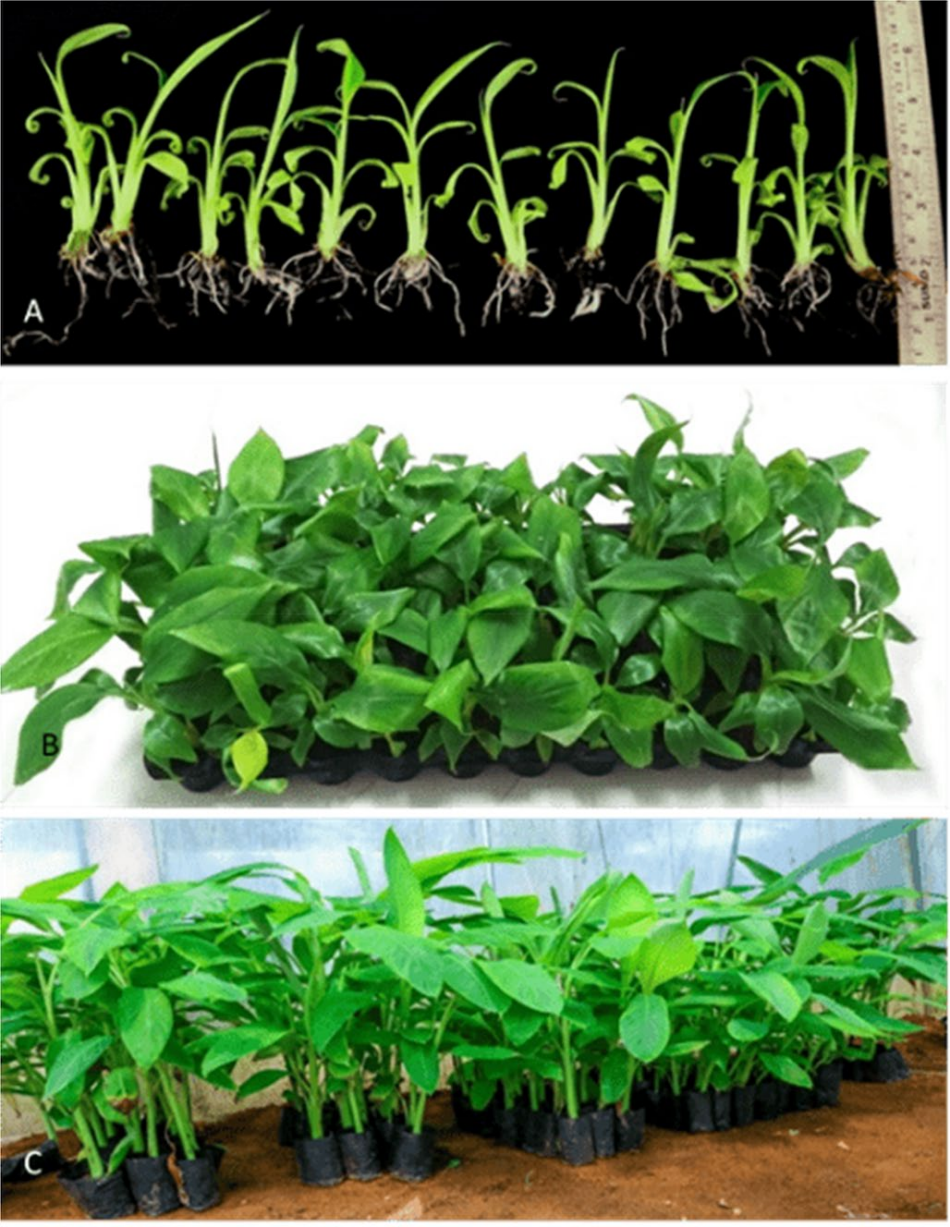
Hardening of plantlets. (**a**) Plantlets produced from bioreactor, (**b**) primary hardened plants, (**c**) SECONDARY hardened plants (Images arranged in Adobe Photoshop CS3).

The survival rate of tissue culture plantlets derived from TIB and SS systems were compared during primary and secondary hardening stages as well as field planting. Significant difference in the survival rate was observed at primary and secondary hardening stages whereas no significant difference was observed at field establishment (Fig. 3). The survival rate of plantlets derived from SS system and TIB in secondary hardening was 84.3% and 95.3% respectively, while at primary hardening 93.6% in SS and 95.3% in TIB and in field conditions 96.6% in SS and 98.3% in TIB (Fig. 4).

### Analysis of photosynthetic pigments, stomatal index and closed stomata

**Figure 5:**
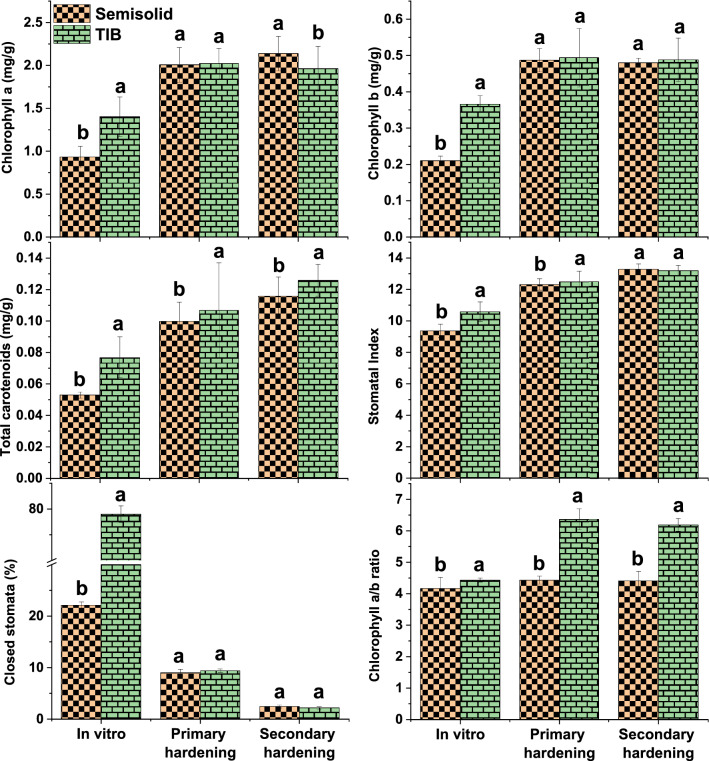
Change in chlorophyll a, b and ratio, carotenoid content, stomatal index and number of closed stomata in leaves of banana plantlets during the harvest from in vitro culture, primary and secondary hardening. (**a**) Chlorophyll a, (**b**) Chlorophyll b, (**c**) Chlorophyll a/b ratio, (**d**) Total carotenoid, (**e**) stomatal index, (**f**) Closed stomata (Graphs were generated using OriginLab Professional Version 2021b software; URL link: www.OriginLab.com/2021b).

The synthesis of photosynthetic pigments and density of stomata in leaves sampled from in vitro, primary and secondary hardened plants grown in TIB and SS system were evaluated to determine photosynthetic functions and results are presented in Fig. 5. Chlorophyll a, b, a/b ratio and carotenoid contents were significantly higher in plants grown in TIB under in vitro condition. During primary hardening, the chlorophyll a and b did not differ significantly in both TIB and SS systems. However, chlorophyll a/b ratio and total carotenoids were significantly higher in TIB than in SS during both primary and secondary hardening. Chlorophyll a content was higher during secondary hardening in semisolid grown plantlets than TIB and no significant change observed in chlorophyll b in both. Stomatal density is a function of both number of stomata and the size of the epidermal cells. Stomatal density is affected mainly by the initiation of stomata and the expansion of epidermal cells (density of epidermal cell), which is a function of variables like light, temperature and water status. The stomatal index in in vitro and primary hardened plants of TIB was significantly higher than SS system and no difference was observed during secondary hardened plants of both TIB and SS systems. Closed stomata prevent water loss, and more number of closed stomata (75%) were observed in TIB grown plant during in vitro conditions than semisolid grown (22.1%), however this number did not vary significantly during primary and secondary hardening of plants from TIB and SS culture conditions.

### ISSR analysis

**Figure 6:**
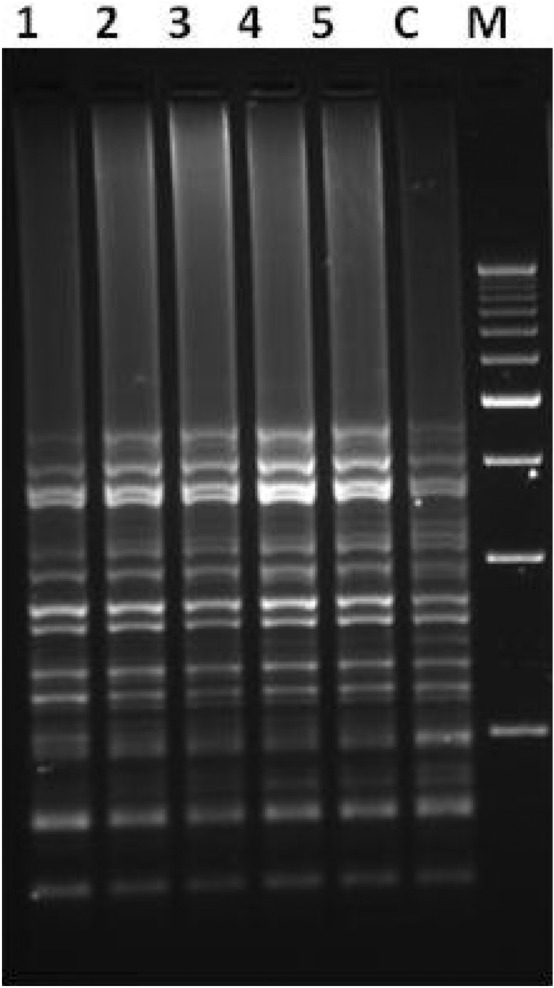
ISSR amplification pattern of plantlets grown in semisolid medium (Lane 1 and 2) and TIB (Lane 3–5) generated using primer (Lane C: Field grown control sample; Lane M: 500 bp Ladder). Leaves sample (randomly chosen 10 different plants pooled as one sample) collected performed the experiment with total of 50 plants.

PCR amplifications using eleven ISSR primers were performed for DNA isolated from leaf samples of plants grown in SS and TIB systems to access genetic integrity. All the primers produced clear and 94 scorable bands ranging in size from 500 to 6000 bp (Table 3). These analyses clearly indicated that there were no polymorphic bands in all the samples processed with the control plants (Fig. 6 and see supplementary file).

**Table 3:** List of ISSR primers used for assessment of genetic fidelity.

Primer name	Sequence 5′ to 3′	Annealing temperature	Total number of bands—polymorphic bands—percent polymorphic bands	
			Semisolid	TIB
UBC807	AGAGAGAGAGAGAGAGT	46.8	12-0-0	12-0-0
UBC808	AGAGAGAGAGAGAGAGC	50.6	8-0-0	8-0-0
UBC810	GAGAGAGAGAGAGAGAT	50.4	6-0-0	6-0-0
UBC811	GAGAGAGAGAGAGAGAC	46.0	6-0-0	6-0-0
UBC812	GAGAGAGAGAGAGAGAA	48.8	11-0-0	11-0-0
UBC818	CACACACACACACACAG	53.0	5-0-0	5-0-0
UBC834	AGAGAGAGAGAGAGAGYT	54.0	7-0-0	7-0-0
UBC836	AGAGAGAGAGAGAGAGYA	51.0	7-0-0	7-0-0
UBC840	GAGAGAGAGAGAGAGAYT	54.0	10-0-0	10-0-0
UBC841	GAGAGAGAGAGAGAGAYC	46.6	5-0-0	5-0-0
UBC842	GAGAGAGAGAGAGAGAYG	48.0	5-0-0	5-0-0

## Discussion and conclusion

The use of temporary immersion system has been reported to improve the shoot multiplication in banana^18,19,21^and other monocot crops such as sugarcane^26^, pineapple^27^ and date palm^28^. There are different types of temporary immersion bioreactor systems available commercially (RITA®, SETIS™, Ebb-and-Flow, TIB®, and MATIS®) and have been utilized for in vitro propagation of banana plants^18,19^. The culture parameters such as immersion time, frequency, volume of media and inoculum density play a significant role in shoot multiplication, As the nutrient uptake, gas exchange and hyperhydricity control varies among species^21,29,30^, the culture parameters need to be standardized.

**Figure 7:**
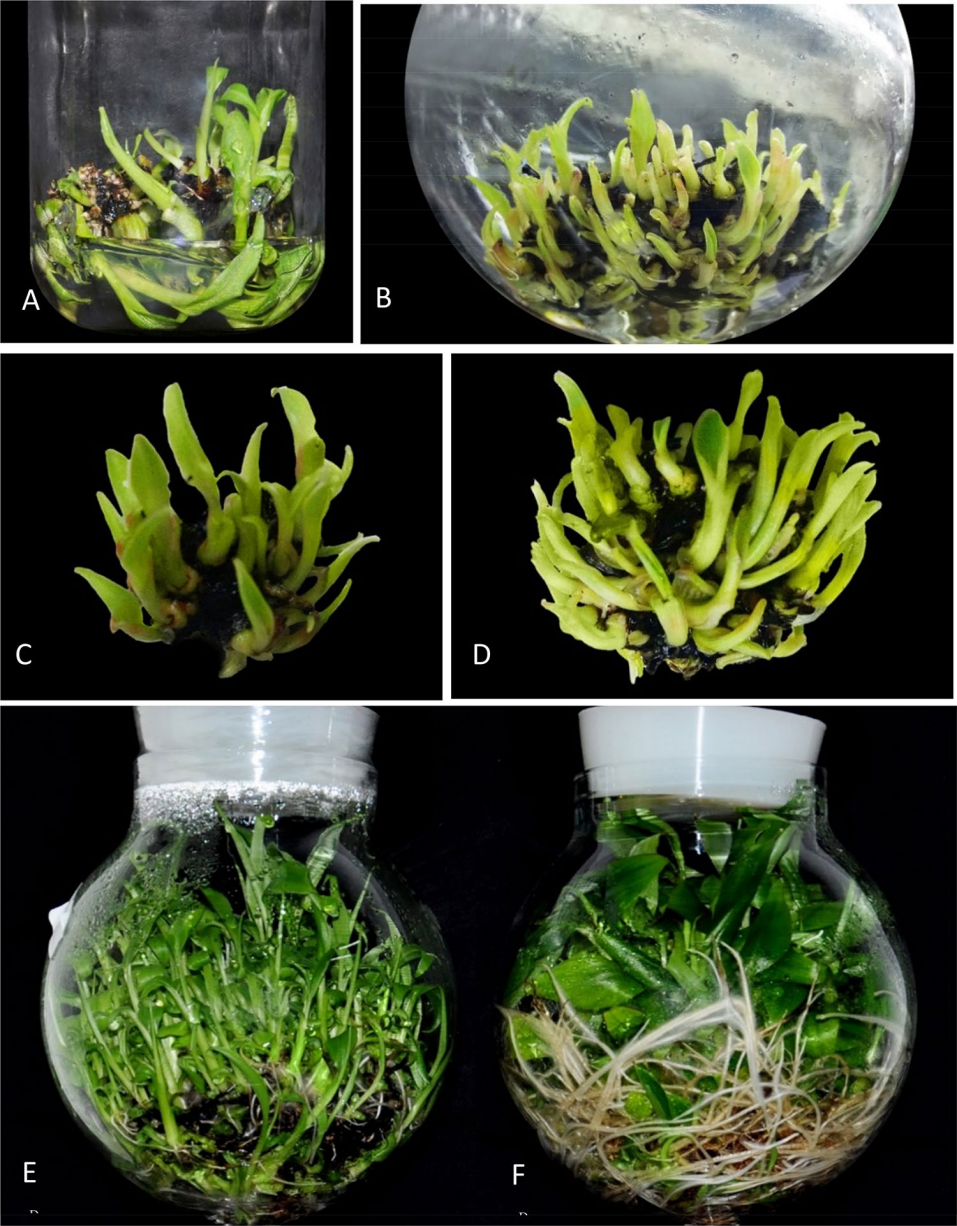
Micropropagation of banana cv. Rasthali in temporary immersion bioreactor. (**A**) Multiple shoots in semisolid culture system. (**B**) Multiple shoots in TIB system. (**C**) Multiple shoot cluster grown in semisolid medium. (**D**) Multiple shoot cluster grown in TIB system. (**E**) Mass multiplied plantlets in TIB system. (**F**) Rooted plantlets in TIB (Images arranged in Adobe Photoshop CS3).

An indigenous temporary immersion bioreactor system has been developed and the protocol has been optimized for shoot multiplication using the explants of 3rd subculture from semi-solid medium (Fig. 7). The explants cultured in TIB with 250 ml of culture medium in 2-min immersion frequency for every 6 h was found to be efficient for shoot multiplication. As reported earlier the most decisive parameter for shoot proliferation includes duration of immersion and volume of nutrient medium^31^. Roels et al.^21^ and Roels et al.^22^ stated that 10 ml of medium per explant influenced the shoot multiplication in plantain banana. Ayub et al.^32^ observed that an optimum volume of culture medium significantly increases shoots per explant in micropropagation of blackberry. Ramos-Castellá et al.^33^ described that 25 ml of media per explant increases the shoot multiplication in *Vanila planifolia.* Zhang et al.^34^ reported that the plantlet growth and morphology is substantially influenced by inoculum density in bioreactor system developed for *Bletilla striata.*

This TIB system has the capacity to produce 2.7-fold shoot multiplication rate than SS system at the end of the 6th subculture. Roels et al.^21^ observed significant difference in multiple shoot production between subcultures, 5th and 6th were significantly higher than in subcultures 8th, 9th and 10th in plantain banana using temporary immersion system. However, Mendes et al.^35^ reported a decrease in multiplication rate of *Musa* cv. Maçã (AAB) in semisolid medium after the fourth subculture. In the present study, no significant variation was observed in the multiplication rate between the subcultures. However, it should be noted that multiple shoot production will vary significantly among the genotypes.

**Figure 8:**
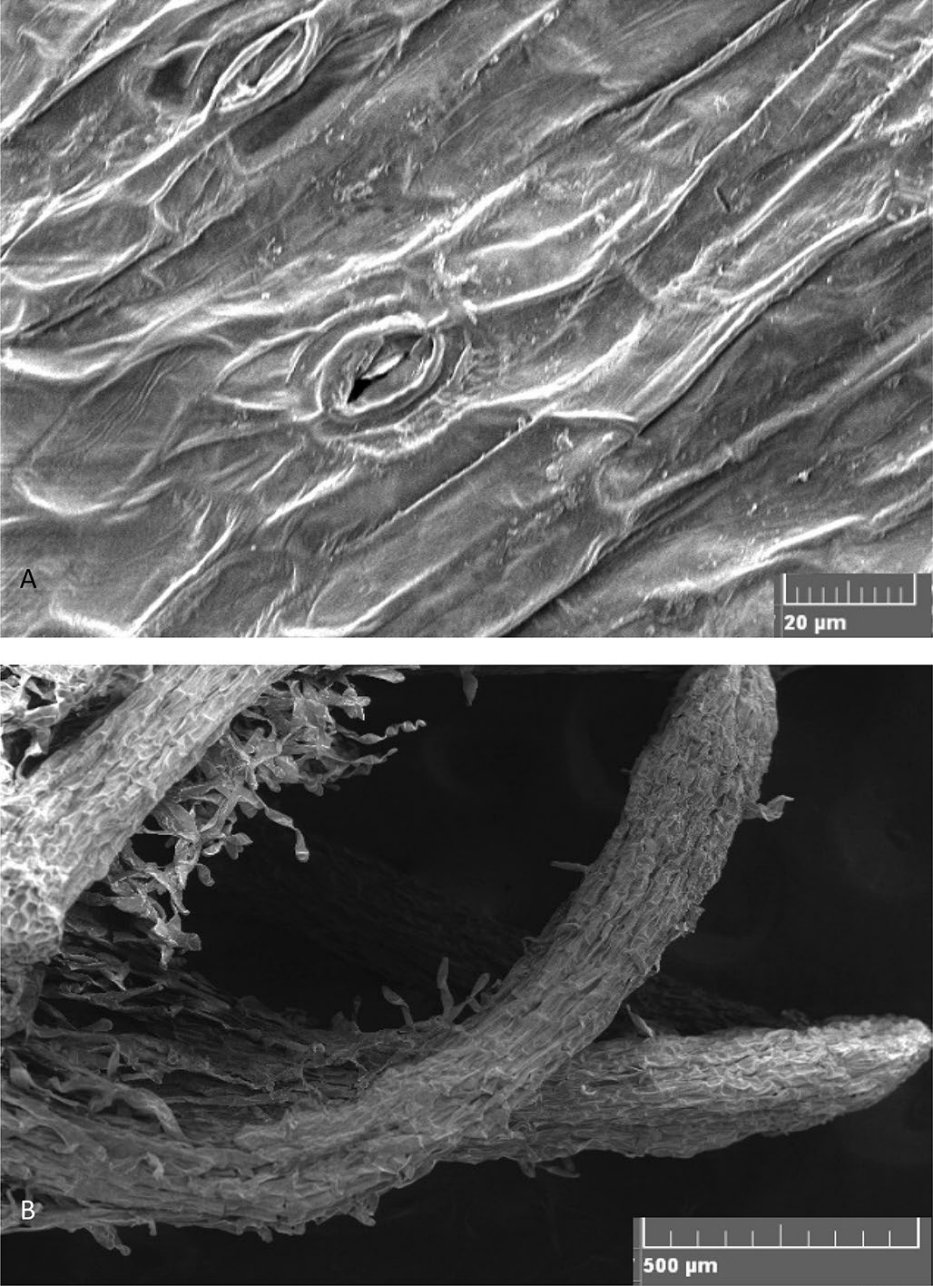
Scanning electron microscopic images. (**A**) Paracytic oblique type of stomata in TIB grown plantlets. (**B**) Lateral roots of TIB grown plants.

TIB system showed superior response in shoot height, root initiation percent, number of roots per shoot and root length. Similar observations were reported in plantain banana, plantlets grown in TIB exhibited better growth, higher photosynthetic rate, improved rooting and longer elongated shoots compared to plantlets grown in semisolid medium^24^. The positive effects of TIB on shoot growth have been demonstrated by many authors in earlier studies^19,36^. In the present study, the immersion frequency of 2 min for every 6 h interval, and 250 ml of rooting medium were found optimum for rooting and shoot elongation. Similarly, Wilken et al.^25^ described, rooting of plantlets with the use of two types of 5 L TIBs with 1 min immersion in every 6 h for cv. Grand Naine was found to be efficient. Contrarily, Bello-Bello et al.^18^ reported that the cv. Grand Naine plantlets survived well without requirement of separate rooting stage. In the present study, rooted shoots were observed during multiplication stage and most of them were only primary roots (Fig. 7F). Absence of lateral roots and root hair formation affect the uptake of nutrients during the acclimatization stage and results in poor survival^37^. The lateral root production ability relies on the interaction of many external and internal factors particularly addition of auxins and other elements of culture media used in the rooting stage^38^. Hence, for the development of primary and lateral roots, the nutrient reservoir was replaced with rooting media and the plantlets produced both primary (Fig. 7F) and lateral roots (Fig. 8B) with adequate root hairs which helped in the survival of the plantlets during the hardening and. Furthermore, the plantlets produced in TIB from all experiments did not show any symptom of phenotypic plasticity, necrosis, chlorosis and hyperhydricity. Rooting efficiency was significantly improved in other crops like hybrid hazelnut grown in temporary immersion system compared to semi-solid medium after four weeks of culture in rooting medium^39^. Other comparable advantages of TIB over in vitro grown plants from semisolid medium, the roots need cleaning to remove the gelling agents often damage the roots and increase the chance of infection. This also contributed for reduced survival rate in the primary hardening stage^40^. TIS has the advantage of reduced the mortality rate at primary hardening.TIB grown plants exhibited higher survival percentage both in primary and secondary hardening with no significant difference in field survival. A similar observation was recorded by several authors for the plantlets obtained from the TIB to that of semisolid culture system^18,23,24,30^.

The physio-chemical parameters of media were recorded to understand the nutrient requirement of the plants grown in TIB^41^. A decrease in pH of the culture medium was observed on 7th day followed by gradual increase. Decrease in pH could be attributed to the release of oxidized phenolic compounds from cut ends of the explants initially, as observed in different liquid and semisolid cultures, and the absorption of NH^4+^ from the medium in exchange with intracellular H^+^^36^. Nevertheless, a steady decrease in EC and TDS was witnessed during shoot multiplication. During shoot elongation and rooting, there was a steady decrease in pH, EC and TDS was observed. Decrease in EC and TDS in TIB grown plants is mainly due to rapid uptake of nutrients at the start of the cycle, coinciding with rapid multiplication of plantlets. However, in the semisolid system, the EC was low at the beginning and increased after 7 days and dropped again, this might be due to binding of nutrients in the gel^42^. A similar phenomenon was reported in micropropagation of cocoyam^41^ and *Eucalyptus* clones grown in RITA® bioreactor^43^.

The success of TIB system not only depends on mass multiplication of plantlets but also depends on the morphological parameters and physiological functions of the plantlets produced^44^. Hence, the synthesis of photosynthetic pigments and density of stomata in leaf samples from in vitro, primary and secondary hardened plants were analysed. Photosynthetic pigment level (a, b, a/b ratio and carotenoid) was significantly higher in TIB than SS system. The design of the TIB permits effective transmission of light, which directly influence the synthesis of photosynthetic pigments^45–47^. The increased carotenoid content in TIB grown plants could be hypothesized as drought response triggered by the periodical supply of liquid nutrient. Carotenoid biosynthesis is regulated by phytoene synthase induced in response to various factors such as light intensity, photoperiod, salt, drought, temperature and abscisic acid synthesis. Drought conditions regulate abscisic acid synthesis and induce roots for survival which might cause increased root length^48–50^. The stomatal indices were found to be significantly high in both in vitro and primary hardened plants of TIB than SS system. Under in vitro conditions, the functionality of stomata is the key physiological process in plant tissues^46^. Stomata are partially functional during in vitro culture condition^51^ and they tend to close their stomata to prevent water loss. In the present study, the number of closed stomata was significantly higher in TIB than semisolid grown plants. Stomata structure and size were studied in leaves of plants grown in TIB and SS system by scanning electron microscope and light microscope (Fig. 8A). There was no difference in stomata structure, size and observed paracytic oblique type of stomata with un-uniform development^52^. In TIB, when the liquid medium come in direct contact with plants along with gaseous atmosphere it stimulates the stomatal activity and contribute the conditioning of plants from in vitro to *ex vitro* conditions and this was reported by several authors^52–56^. Stomata functions are directly correlated to stomata structure and size during the in vitro growth and *ex vitro* survival^51^. However, variation of stomata morphology was also observed under different temporary immersion frequencies tested in *Rhodiola crenulata*^45^.

The sustainability of the large scale micropropagation system mainly depends on the maintenance of the genetic integrity of the plantlets^57^. The occurrence of genetic variation in micro-propagated banana plants mainly due to the use of a higher concentration of growth regulators and an increased number of subcultures as a response to the stress imposed on the plant during the culture period. It has been demonstrated in the form of DNA methylations, chromosome rearrangements and point mutations^16,27,58,59^. To access the genetic uniformity, DNA-based molecular markers of ISSR are widely used^60^. In the present study, only homologous amplifications were observed in samples obtained from both culture systems. Hence, the genetic integrity of plants produced through TIB suggested the suitability of the protocol followed for micropropagation of banana.

**Figure 9:**
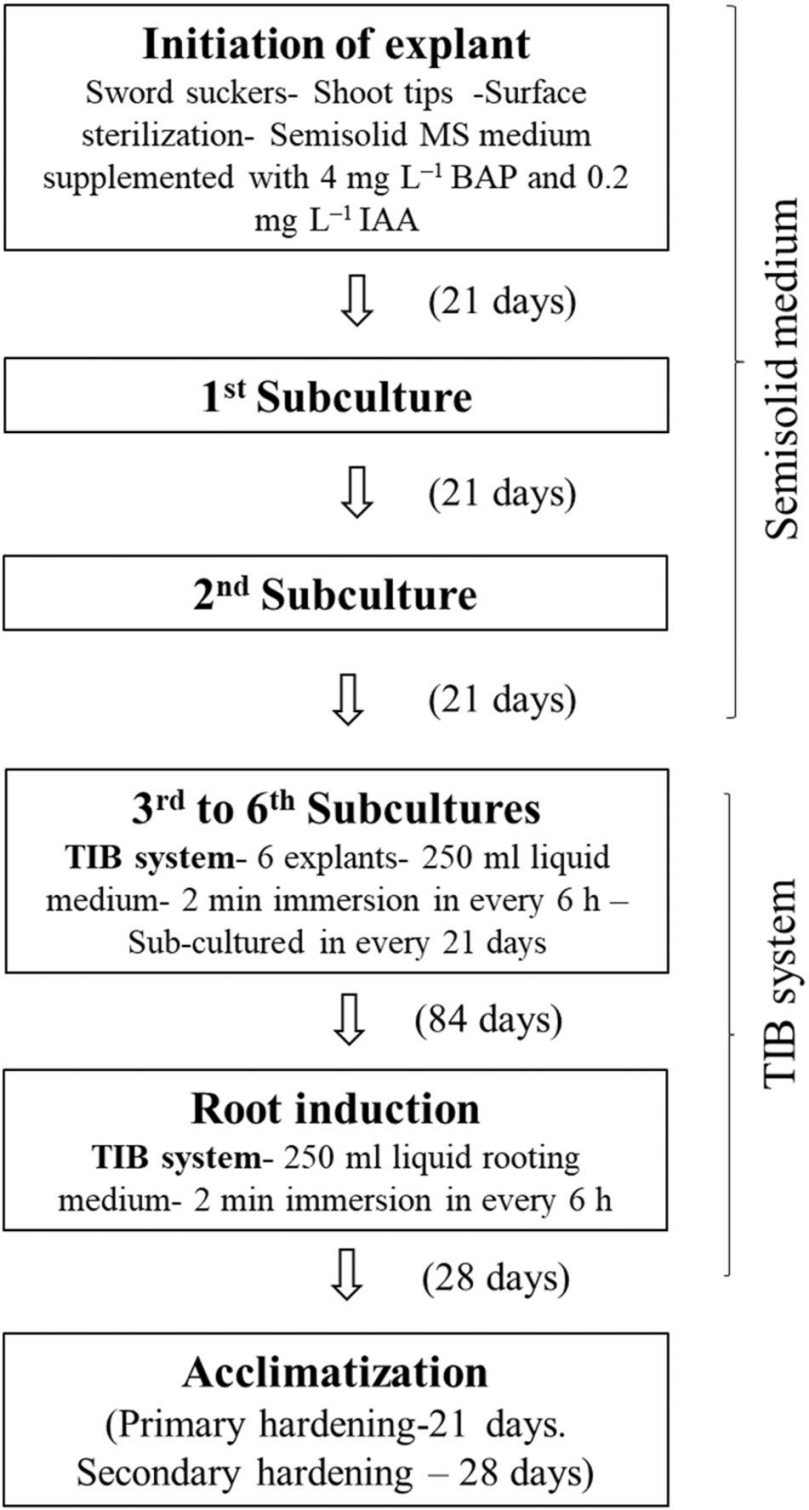
Schematic representation of large-scale in vitro plantlet production in TIB.

This study describes a stable and efficient large scale micropropagation of banana cv. Rasthali (AAB Silk subgroup) in the newly developed TIB system. This system enhanced the shoot multiplication by 2.7 times with a better survival (96%) during acclimatization with a minimum of variation. The plantlets grown in the TIB were genetically uniform. This TIB system with the described protocol (Fig. 9) could be extended to other banana cultivars for commercial/large-scale propagation.

## Materials and methods

### Plant material

In the field gene bank of ICAR-National Research Centre for Banana (NRCB), Tiruchirappalli, Tamil Nadu, India, 372 Indian accessions and 124 exotic accessions are maintained. Being the National Active Germplasm Site (NAGS), all national, international guidelines and legislations are followed in performing the experimental research, field studies and collection of experimental samples. The shoot tip explants were collected from this field gene bank, the IC number allotted for *Musa* sp.cv. Rasthali (AAB Silk subgroup) is 250,750, and the accession number is 0297. The shoot tip explants collected were surface sterilized and cultured on semisolid MS medium^52^ supplemented with 4.0 mg/l 6-Benylamionopurine (BAP) and 0.2 mg/l Indole Acetic Acid (IAA) as described by Saraswathi et al.^61^ after the second sub-culture, the actively proliferating culture were used as explant in TIB system.

### Temporary immersion bioreactor

**Figure 10:**
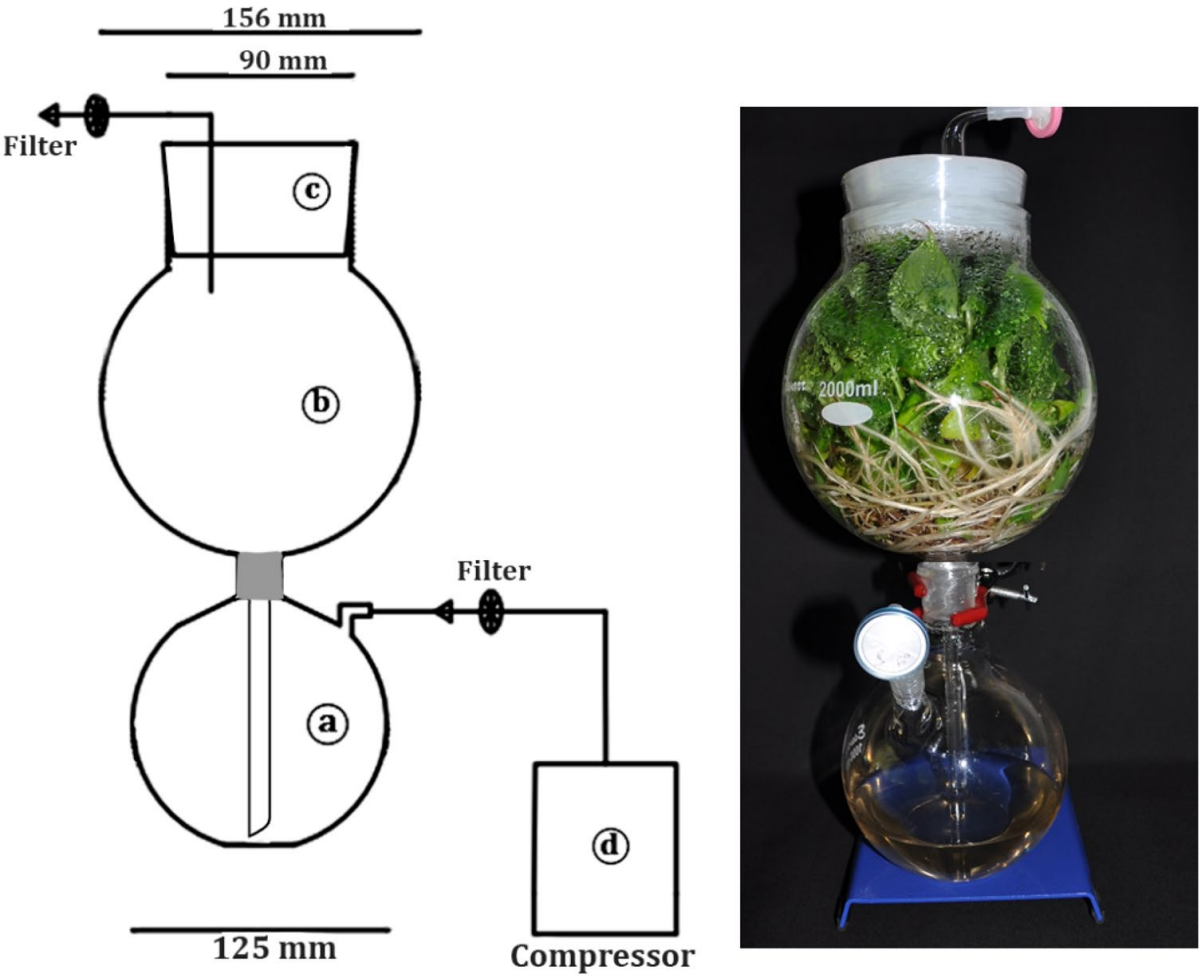
Newly developed temporary immersion bioreactor system. (**a**) Nutrient reservoir, (**b**) growth chamber, (**c**) silicon stopper, (**d**) air compressor. (Line drawing using AutoCAD 2015 version; Image was arranged using Microsoft word).

A new TIB system was developed with two main portions: the upper portion (2000 ml capacity) used as growth chamber is for placing the explants and the lower portion (1000 ml capacity) used as nutrient reservoir for holding the liquid medium (Fig. 10). The nutrient media were supplied periodically to the growth chamber using an air compressor with the help of electronic timer switch.

### Culture systems

The explants were cultured on TIB using MS liquid medium supplemented with 4.0 mg/l BAP, 0.2 mg/l IAA, and 30 g/l sucrose for shoot multiplication. Half strength liquid MS medium supplemented with 1.0 mg/l indole-3-butyric acid (IBA) and 2.0 mg/l 1-Naphthaleneacetic acid (NAA) was used for rooting. The pH was adjusted to 5.8 before autoclaving at 1.2 kg/cm^2^ for 15 min at 120 °C. Primary hardening was carried out using pro-trays containing cocopeat wets with 1/8 MS salts. Secondary hardening was performed using grow-bags containing the mixture of vermicompost and cocopeat (1:1).

The effect of immersion frequency, volume of culture medium and number of explants (inoculum) on multiple shoot induction was examined by growing explants in TIB units containing different volumes of media 100, 250- and 500-ml and different number of explants 3, 6 and 12 for 4 weeks. Cultures were immersed with respective medium for 2 min for every 4, 6, and 12 h. After 21 days, the multiplication medium was replaced by rooting medium and evaluated the morphological characteristics of shoots, synthesis of photosynthetic pigments (chlorophyll a, b and carotenoid content), stomatal index and number of closed stomata to determine the physiological changes in the growth environment. Explants were also cultured on semisolid medium and used as control. All the cultures were maintained at 25 ± 2 °C under a 16/8-h (light/dark) photoperiod with a photosynthetic photon flux of 40 μmol/m^2^/s^1^.

### Shoot multiplication

The shoot tip explants were initiated aseptically and maintained for two subcultures in the semisolid multiplication medium then the proliferating cultures were sub-cultured in multiplication medium using the standardized protocol in TIB (Fig. 10). The cultures were repeatedly sub-cultured at 21 days interval up to 6th passage to examine the effect of subculture on production of shoot buds. During every subculture the number of shoots per explant were recorded for any variation in shoot multiplication. At the 6th subculture shoot buds were harvested and total number of regenerated shoot buds were recorded.

### Physico-chemical parameters of culture media

The media was collected from the bioreactor at regular intervals during the culture period and tested for pH, electrical conductivity (EC) and total dissolved solids (TDS) using a tester (Hanna instruments, USA) to determine the changes in the media components during the course of growth development in TIB.

### Acclimatization of plantlets

After 4 weeks in rooting, the rooted shoots were harvested from the bioreactor unit/culture bottle and the roots were washed in sterile distilled water to remove all traces of media elements. Then the shoots were primary hardened under 80–85% relative humidity (for 21 days). These plantlets were irrigated weekly twice. By the end of three weeks, the plantlets were transferred to net house for secondary hardening (for 28 days).

### Estimation of photosynthetic pigments and, stomatal index

Estimation of photosynthetic pigments (chlorophyll a, b and carotenoid content), stomatal index and number of closed stomata were carried out to determine the physiological functions of the plants grown in bioreactor and semisolid culture. Leaf samples were collected in different stages viz. in vitro (after rooting, at the time of harvest from TIB and SS), primary and secondary hardened stages and processed for the estimation. The chlorophyll content was estimated according to the method of Burnison^62^ with minor modifications. The absorbance was measured at 663 nm (chlorophyll a), 645 nm (chlorophyll b) and 480 nm (carotenoids) using VS 2100 UV–Vis spectrophotometer (Chemito Instruments Pvt. Ltd.).

$$ {\text{Chlorophyll}}\;{\text{a}}\;\left( {{\text{mg}}/{\text{g}}\;{\text{tissue}}} \right) = \left( {{12}.{7}\left( {{\text{A663}}} \right) - {2}.{69}\left( {{\text{A645}}} \right)} \right) \times \frac{v}{{1000{ } \times w}} $$

$$ {\text{Chlorophyll}}\;{\text{b}}\;\left( {{\text{mg}}/{\text{g tissue}}} \right) = \left( {{22}.{9}\left( {{\text{A645}}} \right) - {4}.{68}\left( {{\text{A645}}} \right)} \right) \times \frac{v}{{1000{ } \times w}} $$

$$ {\text{Carotenoid}}\;\left( {{\text{mg}}/{\text{g tissue}}} \right) = \left( {{\text{A48}}0 - 0.{144}} \right) \times \, \left( {{\text{A663}}} \right) - \left( {0.{638}} \right) \times \left( {{\text{A645}}} \right) \times \frac{v}{1000 \times w} $$

Estimation of stomatal index and number of closed stomata were carried out as described byXu and Zhou^63^. The abaxial epidermis of the leaf was cleaned and smeared with nail varnish in the mid area of the leaf and dried at room temperature for 20 min. The thin film was peeled off from the leaf surface and mounted on a glass slide, immediately covered with a cover slip with slight pressure. Number of stomata (s) and epidermal cells (e) for each slide were counted under optical microscope with 10 × and 40 × view. The total number of stomata, numbers of closed stomata per square millimetre were counted. The leaf stomatal index (SI) was estimated using the formula

$$SI=\left(\frac{s}{(s+e)}\right)*100$$

### Sample preparation for scanning electron microscopy

The sampled leaves were prepared for SEM analysis as described by Zakaria and Razak^64^, with minor modifications. Fresh leaves were collected from the in vitro grown plants and fixed immediately in Bouin’s fixative^65^. The leaf samples were kept under vacuum for 4 h and then washed with 1% cacodylate buffer. Post-fixation was done using 1% cacodylate buffered Osmium tetroxide for 2 h. After fixation, samples were dehydrated with graded series of ethanol to absolute ethanol (50% ethanol for 5 min, 70% ethanol for 30 min, 90% ethanol for 30 min, absolute alcohol for 30 min, and each step in two changes at room temperature of 26 ± 2 °C and dried). The dehydrated samples were mounted onto aluminium stubs, coated with a thin layer of gold in an auto sputter fine coater (JFC 1600, Jeol, Japan) and visualized using Scanning Electron Microscope (VEGA-TESCAN-LMU).

### Inter-simple sequence repeat (ISSR) analysis

The genomic DNA from TIB system and semisolid medium grown plantlets were isolated by CTAB method using the protocol of Gawel and Jarret^66^ with minor modifications. The concentration of DNA was measured using ColibriMicrovolume spectrophotometer (Titertek-Berthold, Germany). Prior to PCR amplification, the DNA integrity was checked using 0.8% agarose gel electrophoresis. The genetic stability analysis was performed with 11 ISSR primers (Table 3).

ISSR reaction was carried out with 25 µl PCR reaction performed in a LabnetMultigene™ Opt Max gradient master cycler (Labnet International Inc., Edison, NJ, USA) containing 187.5 ng of DNA, 12.5 µl of Taq 2 × master mix RED (Ampliqon, Denmark) with 25 pmol of different primers**.** ISSR amplification was programmed for initial denaturation at 94 °C for 5 min, followed by 40 cycles of denaturation at 94 °C for 0.30 min, annealing at primer-specific temperatures for 0.30 min, extension at 72 °C for 2 min and final extension at 72 °C for 10.0 min. All the PCRs were repeated three times, using the same conditions to check the accuracy of the amplified products. Amplified products were electrophoresed in 1.5% agarose gel containing 0.25 μg/ml ethidium bromide (Invitrogen, Carlsbad, CA, USA) using 1 × TAE (Tris Acetate EDTA) buffer along with 500 bp ladder. The profile was visualized under UV transilluminator and then documented using gel documentation system (Medicare GELSTAN), only clear and scorable DNA bands were considered for the analysis.

### Statistical analysis

All the experiments were laid out in a completely randomised design with six replicates each and repeated thrice. The data obtained were processed statistically with IBM SPSS Statistics (Version 21) software by one-way analysis of variance (ANOVA) and DMRT (p ≤ 0.05) was performed.

## Supplementary Information

Supplementary Information.
