# Supplementary material for: A novel temporary immersion bioreactor system for large scale multiplication of banana (Rasthali AAB—Silk)
Source: Sci Rep. 2021 Oct 13;11:20371. doi: 10.1038/s41598-021-99923-4 (PMC8514489; doi:10.1038/s41598-021-99923-4)
Supplement: Supplementary file 1 — Supplementary Information. [file 41598_2021_99923_MOESM1_ESM.pdf]

A novel temporary immersion bioreactor system for large scale multiplication of banana (Rasthali AAB - Silk)

Subbaraya Uma[#1*], Raju Karthic[#2], Sathiamoorthy Kalpana[2], Suthanthiram Backiyarani[2], and Marimuthu Somasundaram Saraswathi[2]

Authors' affiliation:
[1]Director, ICAR-National Research Centre for Banana, Thogamalai Main Road, Thayanur Post, Tiruchirappalli, 620 102, Tamil Nadu, India
[2]Bioreactor Facility, Crop Improvement Division, ICAR-National Research Centre for Banana, Thayanur - 620 102, Tiruchirappalli-District, Tamilnadu. India.
[*]Corresponding author Email: umabinit@yahoo.co.in
# these author contributed equally to this work

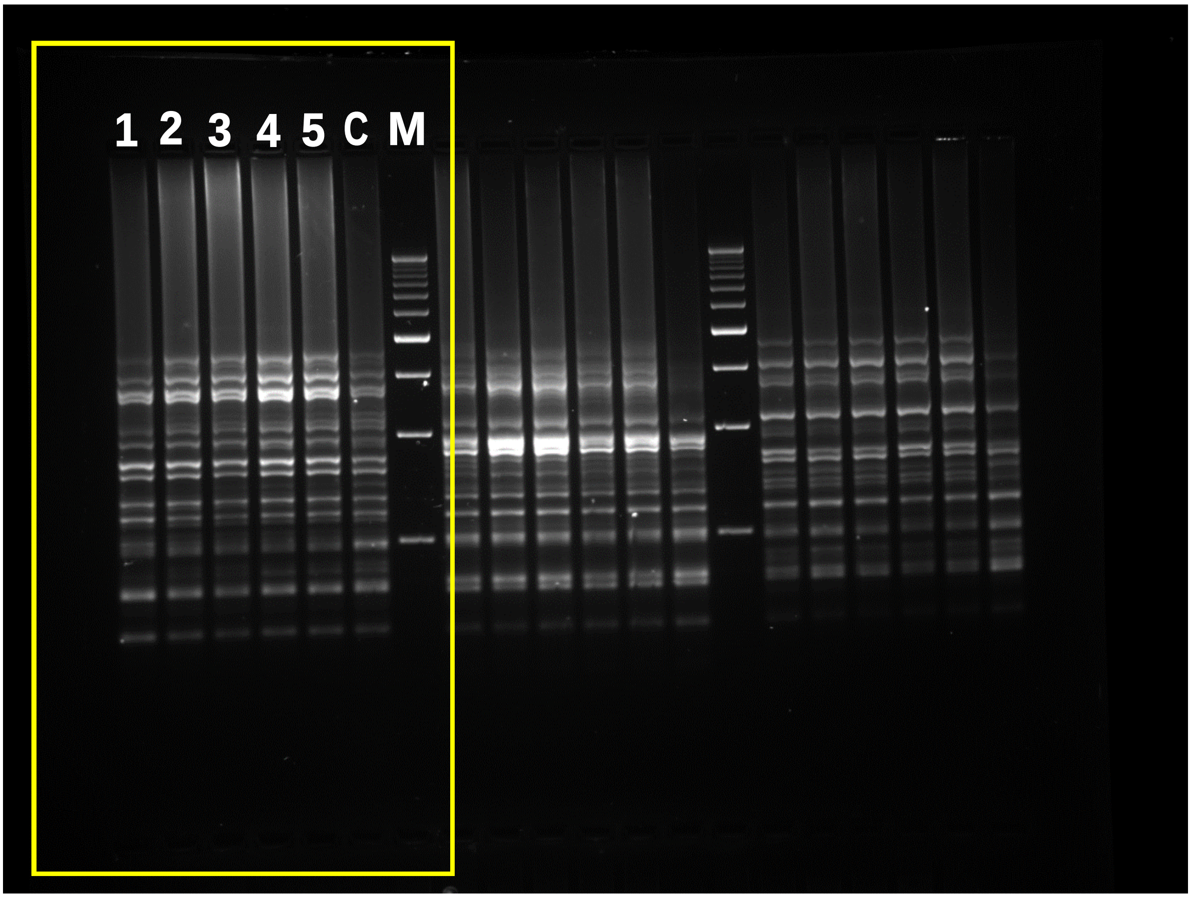

Highlighted area is for Rasthali
